# The New Year

**Published:** 1875-01

**Authors:** N. S. Davis


The New Year.—As the bell tolls out each parting year and rings in the new, the wheels of time appear to us to acquire accelerated speed, and to demand of us sharper, quicker work to avoid falling behind in the race of life. It seems but as yesterday since, with beardless face and timid thought, we dropped the hoe, the scythe, the flail, and with hesitating step left the old stony farm, and wended our way to the country doctor's office, to take the first medical book in hand. And yet the knell of departed time and the happy ring of the new year have greeted us more than forty times since that day. Then two leading thoughts occupied our attention, and became formulated in the question, How can we accomplish the most real good in this life, and at the same time acquire an honorable position among men? To practially solve this question has been the work of the last forty years; and yet, to-day, in turning the thoughts inward for honest, self-examination, we find the same question still prompting and controlling nearly all our work. Whether we have succeeded or failed, we leave for others to decide. This much we know, however, that while sitting here in the last lingering hours of the year 1874, and looking back over the past, we find but little to regret, and nothing of which we would complain. In the whole record, we cannot find an hour devoted to sensual indulgence; not even one hour given up to the lulling influence of tobacco or the exhilarating charms of the wine cup.

We have neither tarried with the sluggard in the arms of Morpheus, nor wasted time and thought in idle wishes and unexecuted resolutions; nor sat down by the wayside to whine, because some one of our brethren was outstripping us in the race of life, or because the world did not duly appreciate ourselves or our profession. We have never prostituted our thoughts for a moment in plotting to circumvent a suppossd enemy, or in organizing rings or factions, either professionally or socially, for our own aggrandizement. Neither can we think of a single human being to-day to whom we would not do an act of kindness were the opportunity afforded us. In truth, the effort to solve the question that occupied our thoughts at the outset has left us no time for self-indulgence, for grumbling, for childish repining, for jealousy of cotemporaries, or even to refuse to do a good deed because it might possibly promote the interests of a supposed rival. To combat vice and promote virtue; to help the poor; to sustain the weak; to lift up the fallen; to alleviate the suffering of the sick; and to improve, sustain and defend the social and educational interests of our profession, have not only been sufficient to absorb all our time and thought, but also to compel a literal obedience to the maxim, "whatsoever thy hands find to do, do it with thy might." Now, reader, if you are in the morning of your professional career, as you enter upon the threshold of this, the last quarter of the nineteenth century, and the chimes that welcome the dawn of 1875 are still lingering in your ears, take up the same question that occupied our thoughts when we first resolved to enter our noble profession; turn it over, scan its bearings, see what a grand central beacon or guide it forms around which to gather the work of a life-time.

If kept steadily before you, it will prompt to every good word and work; fill the world's landscape with green oases, and life's past with precious memories, leaving no room for vain regrets, or time for puerile pleasures or repinings.—*N. S. D., in Medical Examiner.*

